# Expression of MAGE-1 and -3 genes and gene products in human hepatocellular carcinoma

**DOI:** 10.1038/sj.bjc.6690810

**Published:** 1999-11

**Authors:** K Kariyama, T Higashi, Y Kobayashi, K Nouso, H Nakatsukasa, T Yamano, M Ishizaki, T Kaneyoshi, N Toshikuni, T Ohnishi, K Fujiwara, E Nakayama, L Terracciano, G C Spagnoli, T Tsuji

**Affiliations:** The First Department of Internal Medicine andDepartment of Parasitology and Immunology, Okayama University Medical School, 2-5-1 Shikata-cho, Okayama-city, Okayama 700-8558, Japan; The Department of Pathology, University of Basel, Switzerland; The Department of Surgery, Research Laboratories, University Hospital, Basel, Switzerland

**Keywords:** tumour-rejection antigen, cancer testis antigen, immunohistochemistry, Western blotting, immunotherapy

## Abstract

MAGE gene family encodes peptides recognized by autologous cytotoxic T lymphocytes in a major histocompatibility complex (MHC) class-I restricted fashion. In the present study, we have performed reverse-transcription polymerase chain reaction (RT-PCR) for the genes, as well as immunohistochemical analysis and Western blotting of MAGE-1 and -3 proteins in 33 surgically resected hepatocellular carcinomas (HCCs). MAGE-1 and -3 mRNAs were constitutively expressed exclusively in 78 and 42% of HCCs respectively. On immunohistochemistry with monoclonal antibodies, 77B for MAGE-1 and 57B for MAGE-3, MAGE-1 and -3 proteins were recognized in cytoplasm of only six among 33 (18%) and two of 29 HCCs (7%) respectively. The distribution pattern was mostly focal in HCC nodules. By contrast, the Western blot analysis revealed that the MAGE-1 (46 kDa) and -3 proteins (48 kDa) were expressed in 80 and 60% of 15 HCCs examined respectively. The proteins of MAGE-1 and -3 were also expressed exclusively in HCCs regardless of the histological grading and clinical staging. Our results indicate that the detection of the genes by RT-PCR or the proteins by Western blotting is useful for differentiating early HCCs from non-cancerous lesions, and that the peptides derived from MAGE-1 and -3 proteins might be suitable targets for immunotherapy of human HCC. © 1999 Cancer Research Campaign


*British Journal of Cancer* (1999) **81**(6), 1080–1087
© 1999 Cancer Research Campaign
Article no. bjoc.1999.0810

### Expression of MAGE-1 and -3 genes and gene products in human hepatocellular carcinoma

**K Kariyama[1], T Higashi[1], Y Kobayashi[1], K Nouso[1], H Nakatsukasa[1], T Yamano[1], M Ishizaki[1], T Kaneyoshi[1], N Toshikuni[1], T Ohnishi[1], K Fujiwara[1], E Nakayama[2], L Terracciano[3], GC Spagnoli[4] and T Tsuji[1]**

[1]The First Department of Internal Medicine and [2]Department of Parasitology and Immunology, Okayama University Medical School, 2-5-1 Shikata-cho, Okayama-city, Okayama 700-8558, Japan; [3]The Department of Pathology, University of Basel, Switzerland; [4]The Department of Surgery, Research Laboratories, University Hospital, Basel, Switzerland

**Summary** MAGE gene family encodes peptides recognized by autologous cytotoxic T lymphocytes in a major histocompatibility complex (MHC) class-I restricted fashion. In the present study, we have performed reverse-transcription polymerase chain reaction (RT-PCR) for the genes, as well as immunohistochemical analysis and Western blotting of MAGE-1 and -3 proteins in 33 surgically resected hepatocellular carcinomas (HCCs). MAGE-1 and -3 mRNAs were constitutively expressed exclusively in 78 and 42% of HCCs respectively. On immunohistochemistry with monoclonal antibodies, 77B for MAGE-1 and 57B for MAGE-3, MAGE-1 and -3 proteins were recognized in cytoplasm of only six among 33 (18%) and two of 29 HCCs (7%) respectively. The distribution pattern was mostly focal in HCC nodules. By contrast, the Western blot analysis revealed that the MAGE-1 (46 kDa) and -3 proteins (48 kDa) were expressed in 80 and 60% of 15 HCCs examined respectively. The proteins of MAGE-1 and -3 were also expressed exclusively in HCCs regardless of the histological grading and clinical staging. Our results indicate that the detection of the genes by RT-PCR or the proteins by Western blotting is useful for differentiating early HCCs from non-cancerous lesions, and that the peptides derived from MAGE-1 and -3 proteins might be suitable targets for immunotherapy of human HCC. © 1999 Cancer Research Campaign

**Keywords:** tumour-rejection antigen; cancer testis antigen; immunohistochemistry; Western blotting; immunotherapy

MAGE gene family encodes peptides recognized by autologous cytotoxic T lymphocytes in a MHC class-I restricted fashion (van der Bruggen et al, 1991; Marchand et al, 1993; Gaugler et al, 1994) and can elicit tumour cytolytic activity in patients with advanced melanoma (Valomori et al, 1997). De Plaen et al documented that the MAGE gene family, MAGE-A, consists of 12 genes which locate in the chromosome Xq terminal region (De Plaen et al, 1994). Recent investigations have identified new MAGE genes, MAGE-B and MAGE-C1, which appear to be located on chromosome Xp21 and Xq26 (Lurquin et al, 1997; Lucas et al, 1998). MAGE-1 and -3 belong to MAGE-A gene family, and are expressed in a significant proportion of tumours of various histological malignancies, and are silent in normal somatic cells except male germline cells (Weynants et al, 1994; Eura et al, 1995*a*, 1995*b*; Inoue et al, 1995; Patard et al, 1995; Russo et al, 1995; Shichijo et al, 1995; Toh et al, 1995; Yamada et al, 1995; Corrias et al, 1996; Mori et al, 1996; Muramoto, 1997; Sudo et al, 1997). Among the MAGE-A gene family, by contrast, MAGE-11 is highly conserved in mammalian cells of different species, suggesting an important function (Jurk et al, 1998). However, the functions of the MAGE gene family have not been clarified to date.

Analysis of MAGE-1 and -3 gene products have shown that the molecular weight of the former is 46-kDa while that of the latter is 48-kDa (Chen et al, 1994; Schultz-Thater et al, 1994; Kocker et al, 1995; Carrel et al, 1996; Gudat et al, 1996; Gunther et al, 1997). Immunohistochemical studies in malignant melanomas have demonstrated that MAGE-1 gene product is a cytoplasmic protein clustered in paranuclear organelle-like structures (Schultz-Thater et al, 1994). MAGE-3 protein also exists in cytoplasm showing homogeneous, focal or scattered pattern of expression, and the expression undergoes a substantial change in distribution with increase in tumour size and invasiveness (Gunther et al, 1997).

Hepatocellular carcinoma (HCC) is one of the most prevalent malignancies in East Asia, including Japan (Okuda, 1992). Problems to be resolved clinically are how to discriminate well-differentiated HCCs from non-cancerous lesions, and how to suppress the recurrences that frequently occur after treatment. In the present study, we have carried out the reverse transcription polymerase chain reaction (RT-PCR), the immunohistochemical and the Western blot analysis for MAGE-1 and -3 genes and gene products to determine whether detection of the genes, and whether the proteins is available for differential diagnosis of HCC, and whether the peptides derived from MAGE-1 and -3 proteins are suitable targets for the immunotherapy of human HCCs.

### SUBJECTS AND METHODS

We examined 33 surgically resected HCCs. The specimens were cut into slices, formalin-fixed, paraffin-embedded and used for immunohistochemical analysis and for routine staining with haematoxylin and eosin. The samples were also immediately frozen after resection, and stored at –80°C until RNA and protein extraction. The erythroleukemia cell line K562 was used as a

*Received 27 October 1998*
*Revised 11 March 1999*
*Accepted 19 April 1999*

*Correspondence to:* T Higashi

**Table 1**. Clinical and pathological features of the subjects

| Case | Sex | Age(yr) | Bearing liver | Virus type | Tumor size(mm) | Histological grade | Clinical stage | Serum AFP level(ng/ml) |
|---|---|---|---|---|---|---|---|---|
| 1 | M | 66 | CH | C | 18 | well | I | 4 |
| 2 | F | 66 | LC | C | 18 | mod | I | 4818 |
| 3 | M | 70 | CH | C | 18 | mod | I | 3 |
| 4 | F | 62 | LC | C | 20 | well | I | 15 |
| 5 | M | 71 | CH | C | 20 | well | I | 5 |
| 6 | M | 64 | CH | C | 20 | well | I | 17 |
| 7 | M | 65 | LC | C | 20 | well | I | n.d. |
| 8 | M | 68 | LC | NBNC | 20 | mod | I | 14 |
| 9 | M | 57 | CH | C | 27 | well | I | 9 |
| 10 | M | 61 | CH | C | 20 | mod | II | 309 |
| 11 | F | 57 | LC | C | 21 | mod | II | 70 |
| 12 | M | 60 | LC | C | 25 | well | II | 893 |
| 13 | F | 46 | LC | B | 25 | mod | II | 5970 |
| 14 | M | 68 | CH | C | 30 | well | II | n.d. |
| 15 | M | 71 | LC | B | 30 | mod | II | 1443 |
| 16 | M | 71 | CH | C | 30 | mod | II | n.d. |
| 17 | M | 69 | CH | C | 30 | mod | II | 5 |
| 18 | M | 73 | LC | C | 30 | por | II | 0 |
| 19 | M | 64 | CH | C | 35 | well | II | 3 |
| 20 | M | 65 | LC | C | 45 | mod | II | 443 |
| 21 | F | 73 | LC | C | 20 | well | III | 708 |
| 22 | M | 56 | CH | B | 20 | well | III | 10 |
| 23 | F | 68 | LC | C | 20 | mod | III | 1579 |
| 24 | M | 67 | CH | C | 22 | mod | III | 11 |
| 25 | M | 65 | LC | C | 30 | mod | III | 52 |
| 26 | F | 75 | CH | C | 35 | mod | III | 104 |
| 27 | M | 42 | CH | B | 40 | mod | III | 790 |
| 28 | M | 67 | CH | C | 40 | mod | III | 251 |
| 29 | M | 53 | LC | C | 20 | well | IVA | 8 |
| 30 | M | 66 | LC | C | 25 | mod | IVA | 11 |
| 31 | M | 55 | CH | B | 100 | por | IVA | 40000 |
| 32 | F | 68 | LC | C | 12 | mod | IVB | 65 |
| 33 | M | 65 | LC | C | 17 | mod | IVB | 74 |

CH, chronic hepatitis; LC, liver cirrhosis; B, hepatitis B virus; C, hepatitis C virus; NBNC, non hepatitis B virus and non hepatitis C virus; AFP, alfa-feto protein; well, well differentiated hepatocellular carcinoma; mod, moderately differentiated hepatocellular carcinoma; por, poorly differentiated hepatocellular carcinoma; n.d., not done.

positive control of MAGE-1 and -3 gene expression (Serrano et al, 1995), while human testicular tissues were also used as a positive control for the immunostaining and the Western blot analysis (Carrel et al, 1996).

A profile of the 33 patients enrolled in the present study is shown in Table 1. The subjects were 25 males and eight females 42–75 years of age, and five cases were positive for HBs antigen, 27 were positive for hepatitis C virus (HCV) antibody and one was negative for both viral markers. Tumour-bearing non-cancerous tissues were chronic hepatitis in 16 patients and liver cirrhosis in 17 patients. As for tumour size, five were less than 20 mm, 26 were 20–50 mm and one was greater than 51 mm in diameter. The histological grade and clinical stage of HCC were classified according to the criteria outlined by the Liver Cancer Group of Japan (1989). Well, moderately and poorly differentiated HCC comprised 12, 19 and two cases respectively, and stage I, II, III, IVA and IVB accounted for 9, 11, 8, 3 and 2 respectively. The positive rate of alpha-fetoprotein higher than 200 ng/ml was 33%. The study was approved by the institutional review board, and informed consent for the experimental use of specimens was obtained from all patients.

For the detection of MAGE genes, total RNA was extracted using RNAzolB reagent (Tel-Test Inc, Texas, USA) and 2 μg of RNA were used to synthesize cDNA by RAV-2 reverse transcriptase (Takara Biomedics, Osaka, Japan). The sequences of primers for PCR amplification were as follows: MAGE-1; sense, 5′-CGGCCGAAGGAACCTGACCCAG-3′ (CHO-14) and antisense, 5′-GCTGGAACCCTCACTGGGTTGCC-3′ (CHO-12), MAGE-3; sense, 5′-TGGAGGACCAGAGGCCCCC-3′ (AB-1197), antisense, 5′-GGACGATTATCAGGAGGCCTGC-3′ (BLE-5). We also performed RT-PCR for MAGE-4, -6 and -12 to confirm whether the monoclonal antibody against MAGE-3 protein used in the present study detected only MAGE-3 protein since this monoclonal antibody has recently been found to efficiently stain COS cells transfected with MAGE-4, -6 or -12 genes (T Boon, personal communication). The sequences of primers for PCR amplification were as follows: MAGE-4; sense, 5′-ACCAAGGAGAAGATCTGCCAGTGGGTCTC-3′ (MSFf-1) and antisense, 5′-GTCGCCCTCCATTGCATTGTGC-3′ (M41 Sr), MAGE-6; sense, 5′- TGGAGGACCAGAGGCCCCC-3′ (AB-1197), antisense, 5′-CAGGATGATTATCAGGAAGCCTGT-3′ (MAGE-6A). MAGE-12; sense, 5′-AGGTCAGAGAACAGC-GAGAT-3′ (MAGE-12S), antisense, 5′-TTCCTGTTCT-TCGTTGCTGG-3′ (MAGE-12A) (Lee et al, 1996). The PCR reaction was carried out for 35 cycles: 1 min at 94°C and 4 min at 72°C followed by a final extension for 15 min at 72°C for MAGE-1, -3 and -6, and 1 min at 94°C and 1 min at 60°C and 3 min at 72°C followed by a final extension for 15 min for MAGE-4

© 1999 Cancer Research Campaign 

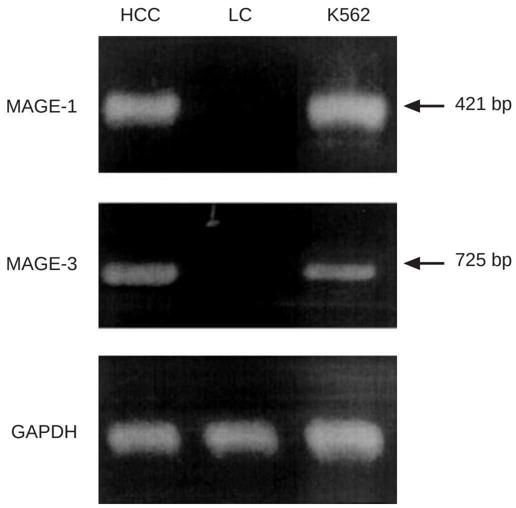

**Figure 1** RT-PCR for MAGE-1 and -3 mRNAs. Total RNA was obtained from a sample of hepatocellular carcinoma (HCC), the corresponding non-cancerous tissue (liver cirrhosis, LC), and K567 cell line. The expected products of 421 and 725 bp which corresponded to MAGE-1 and -3 gene, respectively, were detected in the HCC and K562 cells, but not in the LC.

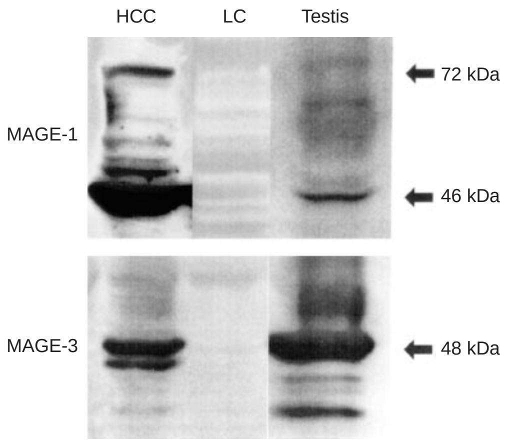

**Figure 2** Expression of MAGE-1 and -3 gene products by western blot analysis using mAbs, 77B for MAGE-1 and 57B for MAGE-3. The molecular weight of MAGE-1 was 46-kDa and that of MAGE-3 protein was 48-kDa, respectively. These proteins were exclusively observed in HCC and the testicular tissue. A 72-kDa band which cross-reacted with the mAb 77B was also detected in two of 15 HCCs. A 31-kDa band was also detected with the mAb 57B only in the testicular tissue.

and -12. The PCR products were electrophoresed on 1% agarose gel and visualized with ethidium bromide staining.

The Western blot analysis was performed in 15 samples. Frozen HCC tissues were homogenized and insoluble cell debris was removed by centrifugation at 12 000 ***g*** for 30 min. Fifty micrograms protein were separated by 12.5% sodium dodecyl sulphate polyacrylamide gel electrophoresis under reducing conditions. The separated proteins were transferred to a nitrocellulose membrane and the membrane was blocked with 5% non-fat dry milk in phosphate-buffered saline (PBS) containing 0.1% Tween-20. Monoclonal antibodies against MAGE-1 (mAb 77B) or MAGE-3 (mAb 57B) were used in the present investigation. To generate these monoclonal antibodies BALB/c mice were immunized with 20 μg of the respective recombinant protein. Hybridoma supernatants were screened by ELISA for binding to the each protein used for immunization (Kocher et al, 1995; Carrel et al, 1996). After overnight incubation with the respective undiluted mAb at 4°C, the membrane was washed with PBS/Tween-20, incubated with horseradish peroxidase (HRP)-conjugated goat anti-mouse second-step antibody (ECL Detection System, Amersham Japan, Tokyo, Japan) for 1 h. Chemiluminescence detection system was used to reveal specific binding (ECL Detection System, Amersham Japan, Tokyo, Japan), and protein bands were visualized on autoradiogrphy film. A commercially available marker of molecular weight (Sigma Chemical Company, Tokyo, Japan) was used.

The immunohistochemical analysis of MAGE-1 and -3 was carried out using the monoclonal antibodies mentioned above. Briefly, 4-μm-sliced sections were deparaffinized and heated in PBS for 10 min at 90°C. After blocking of the endogenous peroxidase activity and non-specific reactivity, the first reaction with the respective monoclonal antibodies was carried out for 1 h at room temperature. Biotin-conjugated anti-mouse immunoglobulin was used as the second antibody after which sections were treated with peroxidase–streptoavidin complex (Nichirei Co Ltd, Tokyo, Japan). The reactive products were visualized by staining with 30% 3,3′-diaminobenzidine tetrahydrochloride (Nichirei Co Ltd, Tokyo, Japan).

### RESULTS

### RT-PCR analysis for the MAGE-1 and -3 mRNAs

The expression of MAGE-1 and -3 genes in case 22 and the K562 cell were shown in Figure 1. The expected products of 421 bp and 725 bp which corresponded to MAGE-1 and -3 gene respectively, were detected in HCC and K562 cells, but not in the non-cancerous tissue (Figure 1).

### Western blot analysis of the MAGE-1 and -3 gene products

The Western blot analysis for MAGE-1 and -3 gene products was performed in 15 HCC and two human testicular tissues. The molecular weight of MAGE-1 and -3 protein was 46-kDa and 48-kDa respectively, and both were clearly distinguishable. The respective gene products were positive exclusively in HCC and testicular tissues, and negative in the corresponding non-cancerous portions. A 72-kDa band, which was reported to be cross-reacted with anti MAGE-1 mAbs (Gudat et al, 1996), was detected in two of 15 HCCs. A 31-kDa band was also observed in immunoblot analysis with mAb 57B. However, this band was only detected in testicular tissues (Figure 2).

### Immunohistochemical studies of the MAGE-1 and -3 gene products

On immunohistochemistry, MAGE-1 protein was recognized in the cytoplasm of six HCC tissues: one showed homogenous and five a focal distribution, of which two exhibited nuclear staining. MAGE-3 protein was also detected in the cytoplasm of two HCC

© 1999 Cancer Research Campaign

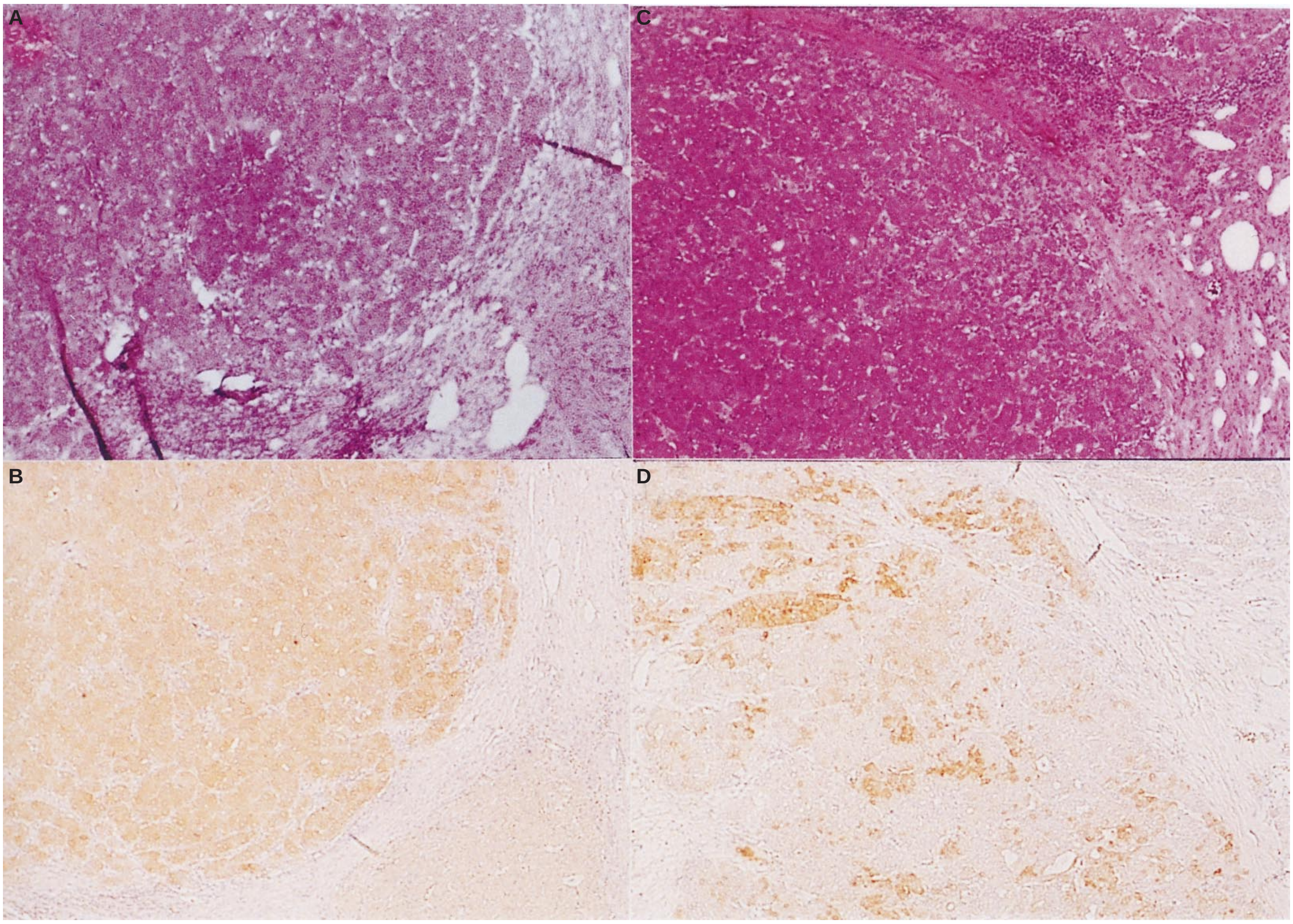

**Figure 3** Immunohistochemical analysis of MAGE-1 and -3 gene products. H-E staining(a)(c). MAGE-1(b) and -3(d) gene products were detected exclusively in the HCC portions. (×40)

tissues. The staining pattern was homogenous or scattered in HCC nodules, and one was positive in the nucleus (Figures 3 and 4).

### mRNA and protein expression of MAGE-1 and -3 and clinicopathological features

The expression of MAGE-1 and -3 gene and gene product in all cases examined was shown in Table 2. The positive rates in the expression of MAGE-1 and -3 gene were 21/27 (77.8%) and 11/26 (42.3%) respectively. Furthermore, their gene products examined by Western blot were positive in 12/15 (80.0%) and 9/15 (60.0%) respectively. However, in the immunohistochemical analysis MAGE-1 and -3 protein was detected only in 6/33 (18.2%) and 2/29 (6.9%) respectively, suggesting that the immunohistochemical analysis was less sensitive than the Western blotting. All of the samples positive in the immunohistochemical examination were positive in the corresponding gene detection analysis, although MAGE-1 and -3 genes were not detected in three samples positive in the Western blot analysis (Table 2). The expression of MAGE-1 and -3 genes and proteins correlated with neither the histological grading nor the clinical staging of HCC (Table 3).

### Expression of MAGE-3, -4, -6 and -12 genes

Since there was a possibility that mAb 57B detected MAGE-4, -6 or -12 except for MAGE-3 protein, we analysed the correspondence between the respective gene and the protein expression. MAGE-4, -6 and -12 mRNA were expressed in 2/9 (22%), 7/9 (78%) and 0/9 (0%). The correspondent rates were 11/14 (79%), 2/9 (22%), 5/9 (56%) and 3/9 (33%) in MAGE-3, -4, -6 and -12 respectively, suggesting that mAb 57B reacted mainly with MAGE-3 gene product rather than the other proteins (Table 4).

### DISCUSSION

Several tumour-associated antigens, including MAGE, BAGE, GAGE families, Melan-A/MART-1, tyrosinase, gp100, have been identified using cytotoxic T-cell clones and T-cell lines isolated from melanoma patients (Bakker et al, 1995; Spagnoli et al, 1995; Marincola et al, 1996). Among these tumour-associated antigens, MAGE-1 and -3 are two clinically relevant and their genes are expressed in a considerable proportion of melanomas and other malignancies. Our result concerning the expression of MAGE-1

*© 1999 Cancer Research Campaign* 

**Table 2.** Expression of MAGE gene and gene products in all cases

| Case | MAGE-1 | | | MAGE-3 | | |
|---|---|---|---|---|---|---|
| No. | mRNA | staining | immunoblot | mRNA | staining | immunoblot |
| 1 | (–) | (–) | (+) | (–) | n.d. | (+) |
| 2 | (+) | (–) | n.d. | (–) | (–) | n.d. |
| 3 | (–) | (–) | n.d. | (–) | n.d. | n.d. |
| 4 | (+) | (–) | n.d. | (–) | (–) | n.d. |
| 5 | (+) | (–) | n.d. | (+) | (–) | n.d. |
| 6 | n.d. | (–) | n.d. | n.d. | (–) | n.d. |
| 7 | (+) | (–) | (–) | (–) | (–) | (–) |
| 8 | (+) | (–) | (+)* | (–) | (–) | (+) |
| 9 | (+) | (–) | n.d. | n.d. | (–) | n.d. |
| 10 | (+) | (+) | (+) | (–) | (–) | (–) |
| 11 | (+) | (+) | n.d. | (+) | (–) | n.d. |
| 12 | (–) | (–) | n.d. | (–) | (–) | n.d. |
| 13 | (+) | (–) | (+) | (–) | (–) | (–) |
| 14 | n.d. | (–) | (+)* | n.d. | (–) | (+) |
| 15 | (+) | (–) | n.d. | (+) | (–) | n.d. |
| 16 | (+) | (–) | (–) | (–) | n.d. | (–) |
| 17 | n.d. | (–) | n.d. | n.d. | (–) | n.d. |
| 18 | (+) | (+) | (+) | (+) | (–) | (+) |
| 19 | n.d. | (–) | n.d. | n.d. | (–) | n.d. |
| 20 | (+) | (–) | n.d. | (+) | (–) | n.d. |
| 21 | n.d. | (–) | n.d. | n.d. | (–) | n.d. |
| 22 | (+) | (+) | (+) | (+) | (+) | (+) |
| 23 | n.d. | (–) | n.d. | n.d. | n.d. | n.d. |
| 24 | (–) | (–) | (+) | (+) | (–) | (+) |
| 25 | (+) | (+) | (+) | (+) | (+) | (+) |
| 26 | (+) | (–) | (+) | (–) | (–) | (–) |
| 27 | (+) | (–) | n.d. | (+) | (–) | n.d. |
| 28 | (+) | (–) | n.d. | (–) | (–) | n.d. |
| 29 | (–) | (–) | (+) | (–) | (–) | (+) |
| 30 | (+) | (+) | (+) | (+) | (–) | (+) |
| 31 | (–) | (–) | (–) | (–) | (–) | (–) |
| 32 | (+) | (–) | n.d. | (–) | (–) | n.d. |
| 33 | (+) | (–) | n.d. | (+) | (–) | n.d. |

n.d., not done;*, positive for band at 72 kDa in Western blot analysis of MAGE-1.

**Table 3**. Positivity of mRNA and protein of MAGE-1 and -3

| | MAGE-1 | | | MAGE-3 | | |
|---|---|---|---|---|---|---|
| | mRNA | Immunoblot | Staining | mRNA | Immunoblot | Staining |
| Histology | | | | | | |
| well | 5/8 | 4/5 | 1/12 | 2/7 | 4/5 | 1/11 |
| mod | 15/17 | 7/8 | 4/19 | 8/17 | 4/8 | 1/16 |
| por | 1/2 | 1/2 | 1/2 | 1/2 | 1/2 | 0/2 |
| Stage | | | | | | |
| I | 6/8 | 2/3 | 0/9 | 1/7 | 2/3 | 0/7 |
| II | 7/8 | 4/5 | 3/11 | 4/8 | 2/5 | 0/10 |
| III | 5/6 | 4/4 | 2/8 | 4/6 | 3/4 | 2/7 |
| IVA | 1/3 | 2/3 | 1/3 | 1/3 | 2/3 | 0/3 |
| IVB | 2/2 | n.d. | 0/2 | 1/2 | n.d. | 0/2 |
| | 21/27 (78) | 12/15 (80) | 6/33 (18) | 11/26 (42) | 9/15 (60) | 2/29 (7) |

(),%; well, well differentiated hepatocellular carcinoma; mod, moderately differentiated hepatocellular carcinoma; por, poorly differentiated hepatocellular carcinoma gene in human HCCs agrees with that previously reported by Yamashita et al (1996). For the induction of MAGE-1 and -3 antigen-specific tumour immunity in HCC patients, the peptide epitopes derived from these antigens should be expressed on the corresponding HLA class I molecules (Celis et al, 1994; van der Bruggen et al, 1994; Yamasaki et al, 1995). Therefore, this study was designed to investigate whether MAGE-1 and -3 proteins are expressed in HCCs, and to determine whether the detection of the genes and proteins is a useful tool for differential diagnosis of HCCs from non-cancerous lesions.

Our findings first provide new information with respect to the expression of MAGE-1 and -3 gene products in human HCCs.

© 1999 Cancer Research Campaign

**Table 4**. Expression of MAGE-4, -6, -12 mRNA and immunoblot analysis with mAb 57B

| Case No. | MAGE-3 mRNA | MAGE-4 mRNA | MAGE-6 mRNA | MAGE-12 mRNA | mAb 57B immunoblot |
|---|---|---|---|---|---|
| 1 | (–) | (–) | (+) | (–) | (+) |
| 7 | (–) | (+) | (+) | (–) | (–) |
| 8 | (–) | (–) | (+) | (–) | (+) |
| 10 | (–) | (–) | (+) | (–) | (–) |
| 13 | (–) | n.d. | n.d. | n.d. | (–) |
| 14 | n.d. | (–) | (+) | (–) | (+) |
| 16 | (–) | n.d. | n.d. | n.d. | (–) |
| 18 | (+) | (–) | (+) | (–) | (+) |
| 22 | (+) | n.d. | n.d. | n.d. | (+) |
| 24 | (+) | (–) | (–) | (–) | (+) |
| 25 | (+) | n.d. | n.d. | n.d. | (+) |
| 26 | (–) | (–) | (+) | (–) | (–) |
| 29 | (–) | (+) | (+) | (–) | (+) |
| 30 | (+) | n.d. | n.d. | n.d. | (+) |
| 31 | (–) | n.d. | n.d. | n.d. | (–) |

n.d., not done.

Monoclonal antibodies 77B and 57B do not show evidence of reciprocal cross-reactivity between MAGE-1 and -3 at the Western level, and they recognize the specific gene products in immunohistochemical sections. However, the 57B mAb has recently been suggested to cross-react with MAGE-4, -6 or -12 genes (T Boon, personal communication) Therefore, we also performed RT-PCR for MAGE-4, -6 and -12 and analysed the correspondence between the expression of the genes and the protein detected by immunoblot analysis with mAb 57B to examine whether the mAb detected only MAGE-3 gene product. As the results, the highly correspondent rate between the gene and the protein in MAGE-3 indicated that mAb 57B detected MAGE-3 protein rather than MAGE-4, -6 and -12, though there was a possibility that the mAb may detect MAGE-6 protein in a few cases.

The Western blot analysis revealed that 80 and 60% of HCCs expressed MAGE-1 and -3 proteins respectively, though the positivities in the immunohistochemistry were lower. This discrepancy might be caused by a lower antigenisity of the proteins in paraffin-embedded samples. With respect to the 72-kDa protein which cross-reacts with the mAb 77B and is frequently co-expressed with the MAGE-1 protein in melanomas (Carrel et al, 1996; Gudat et al, 1996). In the present study, only two of 15 HCCs expressed this protein and these two HCCs also expressed MAGE-1 protein. As the positive rates of MAGE-1 and -3 genes are different among various malignancies (Weynants et al, 1994; Eura et al, 1995*a*, 1995*b*; Inoue et al, 1995; Patard et al, 1995; Russo et al, 1995; Shichijo et al, 1995; Toh et al, 1995; Yamada et al, 1995; Corrias et al, 1996; Mori et al, 1996; Muramoto, 1997; Sudo et al, 1997), the expression of the 72-kDa protein might differ between melanomas and HCCs.

Regarding the protein distribution, our results are consistent with those of prior studies in melanomas showing that MAGE-1 and -3 gene products are immunopositive only in the malignant portions, cytoplasmic proteins invaginating into distorted nuclei, and distribute either homogeneously, focally or singularly (Schultz-Thater et al, 1994; Kocker et al, 1995; Gudat et al, 1996; Gunther et al, 1997).

An apparent discordance between the gene expression and the protein detection of MAGE-1 and -3 has been shown in melanomas: five expressed MAGE-1 protein whereas the gene was detected in four samples, and seven expressed MAGE-3 protein among nine positive for the gene expression (Gudat et al, 1996; Gunther et al, 1997). This discordance was also observed in the present study on HCCs, in which three were positive in the Western blotting despite being negative in the gene expression. Although the protein for the Western blot analysis might be better preserved than the mRNA for RT-PCR, we have no explanation for this discrepancy.

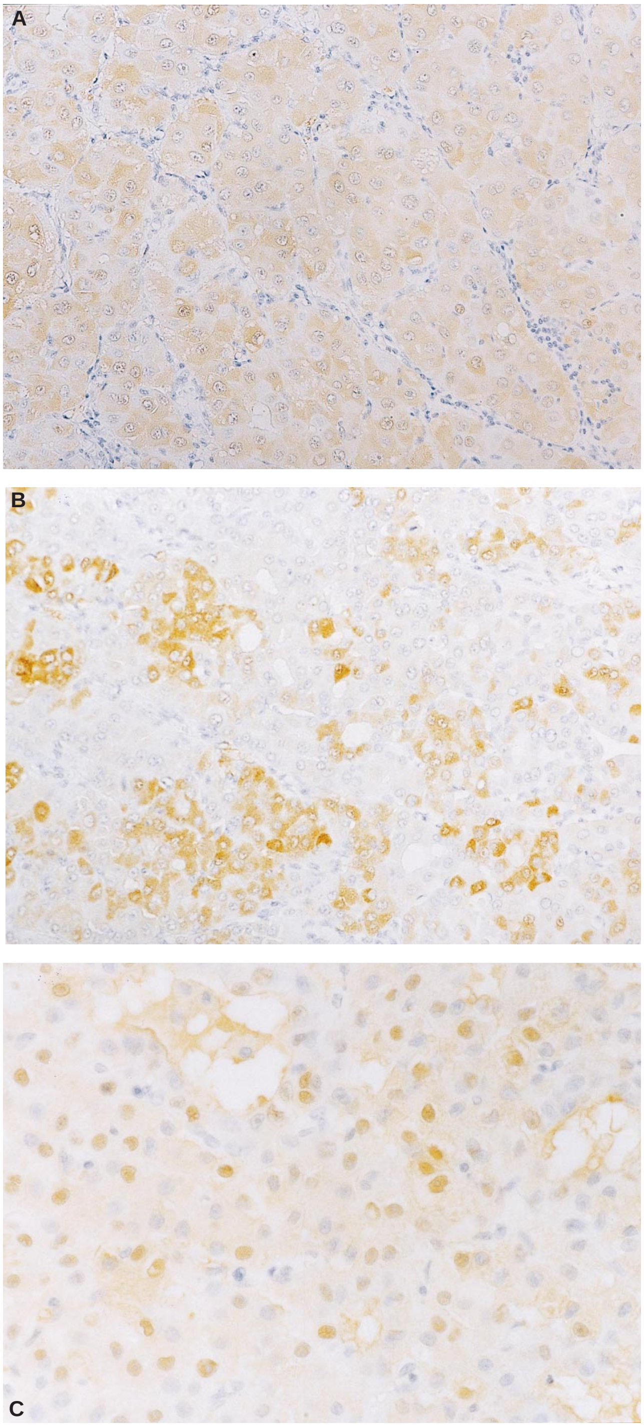

**Figure 4**. MAGE-1 protein was detected in cytoplasm of HCC cells, and the distribution pattern were homogeneous(a) or focal(b). (×200) In some samples, the protein was recognized in the nucleus of HCC cells(c). (×400)

In the present study, we have demonstrated that MAGE-1 and -3 proteins were detected exclusively in HCC areas regardless of the histological grading and clinical staging. The positive rates of MAGE-1 and -3 proteins analyzed by the immunoblotting were higher than those examined by the immunohistochemical analysis. Gene and protein of MAGE-1 were more frequently expressed than those of MAGE-3 in human HCCs. Furthermore, we have recently attempted to detect the MAGE-1 gene in small samples gained by a 21-gauge thin-needle biopsy, and found the constitutive gene expression in early HCCs (solitary and less than 20 mm in diameter) ambiguous in histological examination. Thus, at the present time, the detection of MAGE-1 gene by RT-PCR or gene product by the Western blotting is the best choice for differential diagnosis of early HCCs from non-cancerous lesions.

Collective evidence indicates that MAGE-1 and -3 peptides are recognized by autologous cytotoxic T lymphocytes and can elicit cytolytic activity in a MHC class I-restricted manner in vitro or in vivo in the patients with melanoma or other malignancies (Celis et al, 1994; van der Bruggen et al, 1994; Yamasaki et al, 1995; Hu et al, 1996; Toso et al, 1996; Fleischhauer et al, 1997; Valomori et al, 1997). A strong CTL response was induced in patients with advanced melanoma by vaccination of dendritic cells pulsed with a cocktail of peptides of tumour-associated antigens including MAGE-1 and -3 together with or without cell lysate (Nestlé et al, 1998). The fact that not only the primary, but also metastatic, melanomas were regressed following vaccination suggests the clinical usefulness of the immunotherapy against tumour-associated antigen peptides. Many reports have indicated the possibility of vaccine therapy in malignant tumours with tumour-rejection antigen peptides; however, most of them did not show the protein expression in tumours. We clarified in the present study that the gene products of MAGE-1 and -3 were frequently expressed in HCCs and thus the possibility of immunotherapy against MAGE-1 or -3 epitopes in HCC patients could be envisaged moreover.

In conclusion, we demonstrated the expression of MAGE-1 and -3 genes and gene products in human HCCs. Our findings indicate that the detection of the genes and gene products of MAGE-1 and -3 is a useful tool for differential diagnosis between early HCCs and non-cancerous lesions, and immunotherapy targeting the peptides derived from MAGE-1 and -3 proteins appears to be feasible in patients with HCC.

### ACKNOWLEDGEMENTS

We are grateful to Miss Miho Sasaki for her skilful preparation of tissue sections for immunohistochemical and haematoxylin-eosin staining. This work was supported by grants from Intractable Hepatitis Research Committee, the Japanese Ministry of Health and Welfare and also from the Japanese Ministry of Education (No. 10670474).

### REFERENCES

Bakker AB, Marland G, de Boer AJ, Huijbens RJ, Danen EH, Adema GJ and Figdor CG (1995) Generation of antimelanoma cytotoxic T lymphocytes from healthy donors after presentation of melanoma-associated antigen-derived epitopes by dendritic cells in vitro. *Cancer Res* **55**: 5330–5334

Carrel S, Schreyer M, Spagnoli GC, Cerottini JC and Rimodi D (1996) Monoclonal antibodies against recombinant-MAGE-1 protein identify a cross-reacting 72-kDa antigen which is co-expressed with MAGE-1 protein in melanoma cells. *Int J Cancer* **67**: 417–422

Celis E, Fikes J, Wentworth P, Sidney J, Southwood S, Maewal A, Del Guercio MF and Livingston B (1994) Identification of potential CTL epitopes of tumor-associated antigen MAGE-1 for five common HLA-A alleles. *Mol Immunol* **31**: 1423–1430

Chen Y-T, Stockert E, Chen Y, Garin-Chesa P, Rettig WJ, van der Bruggen P, Boon T and Old LJ (1994) Identification of the MAGE-1 product by monoclonal and polyclonal antibodies. *Proc Natl Acad Sci USA* **91**: 1004–1008

Corrias MV, Scaruffi P, Occhino M, De Bernardi B, Tonini GP and Pistoia V (1996) Expression of MAGE-1, MAGE-3 and MART-1 genes in neuroblastoma. *Int J Cancer* **69**: 403–407

De Plaen E, Arden K, Traversari C, Gaforio JJ, Szikora JP, De Smet C, Brasseur F, van der Bruggen P, Lethe B, Lurquin C, et al (1994) Structure, chromosomal localization, and expression of 12 genes of the MAGE family. *Immunogenetics* **40**: 360–369

Eura M, Chikamatsu K, Ogi K, Nakano K, Masuyama K and Ishikawa T (1995*a*) Expression of MAGE-1, -2, and -3 by human maxillary carcinoma. *Anticancer Res* **15**: 55–59

Eura M, Ogi K, Chikamatsu K, Lee KD, Nakano K, Masuyama K, Itoh K and Ishikawa T (1995*b*) Expression of the MAGE gene family in human head-and-neck squamous-cell carcinomas. *Int J Cancer* **64**: 304–308

Fleischhauer K, Tanzarella S, Russo V, Sensi ML, van der Burggen P, Bordignon C and Traversari C (1997) Functional heterogeneity of HLA-A*02 subtypes revealed by presentation of a MAGE-3-encoded peptide to cytotoxic T cell clones. *J Immunol* **159**: 2513–2521

Gaugler B, Van den Eynde B, Van der Burggen P, Romero P, Gaforio JJ, De Plaen E, Lethe B, Brasseur F and Boon T (1994) Human gene MAGE-3 codes for an antigen recognized on a melanoma by autologous cytolytic T lymphocytes. *J Exp Med* **179**: 921–930

Gudat F, Zuber M, Durmuller U, Kocher T, Schaefer C, Noppen C and Spagnoli GC (1996) The tumor-associated antigen MAGE-1 is detectable in formalin-fixed paraffin sections of malignant melanoma. *Virchows Arch* **429**: 77–81

Gunther FL, Hofbauer L, Schaefer C, Noppen C, Boni R, Kamarashev J, Nestlé FO, Spagnoli GC and Dummer R (1997) MAGE-3 immunoreactivity in formalin-fixed paraffin-embedded primary and metastatic melanoma. *Am J Pathol* **151**: 1549–1553

Hu X, Chakraborty NG, Sporn JR, Kurtzman SH, Ergin MT and Mukherji B (1996) Enhancement of cytolytic T lymphocyte precursor frequency in melanoma patients following immunization with the MAGE-1 peptide loaded antigen presenting cell-based vaccine. *Cancer Res* **56**: 2479–2483

Inoue H, Mori M, Honda M, Li J, Shibuta K, Mimori K, Ueo H and Akiyoshi T (1995) The expression of tumor-rejection antigen 'MAGE' genes in human gastric carcinoma. *Gastroenterology* **109**: 1522–1525

Jurk M, Kremmer E, Schwarz U, Forster R and Winnacker EL (1998) MAGE-11 protein is highly conserved in higher organisms and located predominantly in the nucleus. *Int J Cancer* **75**: 762–766

Kocker T, Schultz-Thater E, Gudat F, Schaefer C, Casorati G, Juretic A, Willimann T, Harder F, Heberer M and Spagnoli GC (1995) Identification and intracellular localization of MAGE-3 gene product. *Cancer Res* **55**: 2236–2239

Lee KD, Eura M, Ogi K, Nakano K, Chikamatsu K, Masuyama K and Ishikawa K (1996) Expression of the MAGE-1, -2, -3, -4, and -6 genes in the non-squamous cell carcinoma lesions of the head and neck. *Acta Otolaryngol* **116**: 633–639

Liver Cancer Study Group of Japan (1989) The general rules for the clinical and pathological study of primary liver cancer. *Jpn J Surg* **19**: 98–129

Lucas S, De Smet C, Arden KC, Viars CS, Lethe B, Lurquin C and Boon T (1998) Identification of a new MAGE gene with tumor-specific expression by representational difference analysis. *Cancer Res* **58**: 743–752

Lurquin C, De Smet C, Brasseur F, Muscatelli F, Martelange V, De Plaen E, Brasseur R, Monaco AP and Boon T (1997) Two members of the human MAGE B gene family located in Xp21.3 are expressed in tumors of various histological origins. *Genomics* **46**: 397–408

Marchand M, Brasseur F, van der Bruggen P, Coulie P and Boon T (1993) Perspectives for immunization of HLA-A1 patients carrying a malignant melanoma expressing gene MAGE-1. *Dermatology* **186**: 278–280

Marincola FM, Rivoltini L, Salagaller ML, Player M and Rosenberg SA (1996) Differential anti-MART-1/MelanA CTL activity in peripheral blood of HLA-A2 melanoma patients in comparison to healthy donors: evidence of in vivo priminig by tumor cells. *J Immunother Emphasis Tumor Immunol* **19**: 266–277

Mori M, Inoue H, Mimori K, Shibuta K, Baba K, Nakashima H, Haraguchi M, Tsuhi K, Ueo H, Barnard GF and Akiyoshi T (1996) Expression of MAGE genes in human colorectal carcinoma. *Ann Surg* **224**: 183–188

Muramoto T (1997) Detection of MAGE-1 tumor antigen in brain tumor. *Kurume Med J* **44**: 43–51

 *© 1999 Cancer Research Campaign*

Nestlé FO, Alijagic S, Gilliet M, Sun Y, Grabbe S, Dummer R, Burg G and Schadendorf D (1998) Vaccination of melanoma patients with peptide- or tumor lysate-pulsed dendritic cells. *Nature Med* **4**: 328–332

Okuda K (1992) Hepatocellular carcinoma: recent progress. *Hepatology* **15**: 948–963

Patard JJ, Brasseur F, Gil-Diez S, Radvanyi F, Marchand M, Francois P, Abi-Aad A, Van Cangh P, Abbou CC and Chopin D (1995) Expression of MAGE genes in transitional-cell carcinomas of the urinary bladder. *Int J Cancer* **64** 60–64

Sudo T, Kuramoto T, Komiya S, Inoue A and Itoh K (1997) Expression of MAGE genes in osteosarcoma. *J Orthop Res* **15**: 128–132

Toh Y, Yamana H, Schichijo S, Fujita H, Tou U, Sakaguchi M, Kakegawa T and Itoh K (1995) Expression of MAGE-1 gene by esophageal carcinoma. *Jpn J Cancer Res* **86**: 714–717

Toso JF, Oei C, Oshidari F, Tartaglia J, Paoletti E, Lyerly HK, Talib S and Weinhold KJ (1996) MAGE-1-spcific precursor cytotoxic T-lymphocytes present among tumor infiltrating lymphocytes from a patient with breast cancer: characterization and antigen-specific activation. *Cancer Res* **56**: 16–20

Valomori D, Lienard D, Waanders G, Rimldi D, Cerottini JC and Romero P (1997) Analysis of MAGE-3-specific cytolytic lymphocytes in human leukocyte antigen-A2 melanoma patients. *Cancer Res* **57**: 735–741

van der Bruggen P, Szikora JP, Boel P, Wildmann C, Somville M, Sensi M and Boon T (1994) Autologous cytolytic lymphocytes recognized a MAGE-1 nonapeptide on melanomas expressing HLA-Cw*1601. *Eur J Immunol* **24**: 2134–2140

van der Bruggen P, Traversari C, Chomez P, Lurquin C, De Plaen E, Van den Eynde B, Knuth A and Boon T (1991) A gene encoding an antigen recognized by cytolytic lymphocytes on a human melanoma. *Science* **254**: 1643–1647

Weynants P, Lethe B, Brasseur F, Marchand M and Boon T (1994) Expression of mage genes by non-small-cell lung carcinomas. *Int J Cancer* **56**: 826–829

Yamada A, Kataoka A, Shichijo S, Imai Y, Nishida T and Itoh K (1995) Expression of MAGE-1, MAGE-2, MAGE-3/-6 and MAGE-4a/-4b genes in ovarian tumors. *Int J Cancer* **64**: 388–393

Yamasaki S, Okino T, Chakraborty NG, Adkisson WO, Sampieri A, Padula SJ, Mauri F and Mukherji B (1995) Presentation of synthetic peptide antigen encoded by the MAGE-1 gene by granulocyte/macrophage-colony-stimulating-factor-cultured macrophages from HLA-A1 melanoma patients. *Cancer Immunol Immunother* **40**: 268–271

Yamashita N, Ishibashi H, Hayashida K, Kudo J, Takenaka K, Itoh K and Niho Y (1996) High frequency of the MAGE-1 gene expression in hepatocellular carcinoma. *Hepatology* **24**(6): 1437–1440

*© 1999 Cancer Research Campaign*
